# Multivariate analysis of genomic variables, effective population size, and mutation rate

**DOI:** 10.1186/s13104-019-4097-3

**Published:** 2019-01-25

**Authors:** Punit Bhattachan, Bo Dong

**Affiliations:** 1 Key Laboratory of Marine Genetics and Breeding, College of Marine Life Sciences, Ocean University of China, No. 5 Yushan Road, Qingdao, 266003 China; 2 Laboratory for Marine Biology and Biotechnology, Qingdao National Laboratory for Marine Science and Technology, Qingdao, 266237 China; 3 Institute of Evolution & Marine Biodiversity, Ocean University of China, Qingdao, 266003 China

**Keywords:** Genomic variables, Multivariate analysis, Genome evolution, Genetic drift

## Abstract

**Objective:**

The relationship between genomic variables (genome size, gene number, intron size, and intron number) and evolutionary forces has two implications. First, they help to unravel the mechanism underlying genome evolution. Second, they provide a solution to the debate over discrepancy between genome size variation and organismal complexity. Previously, a clear correlation between genomic variables and effective population size and mutation rate (*Neu*) led to an important hypothesis to consider random genetic drift as a major evolutionary force during evolution of genome size and complexity. But recent reports also support natural selection as the leading evolutionary force. As such, the debate remains unresolved.

**Results:**

Here, we used a multivariate method to explore the relationship between genomic variables and *Neu* in order to understand the evolution of genome. Previously reported patterns between genomic variables and *Neu* were not observed in our multivariate study. We found only one association between intron number and *Neu*, but no relationships were observed between genome size, intron size, gene number, and *Neu*, suggesting that *Neu* of the organisms solely does not influence genome evolution. We, therefore, concluded that *Neu* influences intron evolution, while it may not be the only force that provides mechanistic insights into genome evolution and complexity.

**Electronic supplementary material:**

The online version of this article (10.1186/s13104-019-4097-3) contains supplementary material, which is available to authorized users.

## Introduction

All eukaryotic genomes possess similar features such as genome size, gene number, intron size, intron number, and transposable elements. These genomic variables can be attributed to the evolutionary forces acting over long evolutionary time. In the previous analysis, genomic variables were shown to have a strong correlation with the effective population size (*Ne*) and mutation rate (*u*) of the organism. Expansion of genome size and complexity were attributed to random genetic drift [1]. This is also known as the mutational hazard hypothesis [2], and it is influential in terms of genome evolution and complexity [3].

The interplay between *Ne* and *u* may impact genome size variation across kingdoms, and can be regarded as a plausible explanation to understand mechanism of genome evolution [2, 4, 5]. On the other hand, many old theories such as mutational pressure, nucleoskeletal, and nucleotypic have emphasized adaptive arguments to explain genome size variation and evolution. But these arguments received minimal recognition [6]. In addition, the mutational equilibrium model stated that each species acquired their own genome size by deletion or insertion, and thus different species showed variation in their genome size [7]. We think that genomic variables being a multivariate dataset of a genome, multivariate statistical analysis is important to examine the relationship between genomic variables and *Neu* in order to understand genome evolution.

## Main text

### Methods

#### Data collection

Data on genomic variables and *Neu* were obtained from reference [1, 8–13, NCBI annotation release101]. Some of the species had missing data on either one of the genomic variables or *Neu*; therefore, we excluded them from the multivariate analysis because it will cause misinterpretation of the dataset. Species were grouped in rows, while genomic variables and *Neu* were represented as columns to create a multivariate dataset (Additional file 1: Table S1).

#### Multivariate statistical analysis

Multivariate analysis called principal component analysis (PCA), cluster analysis (CA), and exploratory factor analysis (EFA) were carried out by executing stats package in R software (R version 3.2.3). The correlation matrix was chosen in the PCA analysis for the dataset (Additional file 1: Table S1). In addition, the dataset was standardized, centered, scaled, and prcomp function was used to perform PCA analysis. A hierarchical cluster analysis was carried out after standardization of the dataset (Additional file 1: Table S1), using euclidean distance method. Then, ward. D2 method in R software was applied to construct dendrogram by utilizing hclust function. The exploratory factor analysis was also performed in R by executing factanal function, which is based on maximum-likelihood methods. In addition, the dataset (Additional file 1: Table S1) was standardized, rotated by varimax, and only two factors were considered during analysis. The test for two factors is sufficient for the hypothesis showed Chi square statistic 1.35 and p-value 0.246 for the raw data, while Chi square statistic 2.32 and p-value 0.128 for the phylogenetically independent contrasts in EFA.

#### Phylogenetically independent contrasts (PICs) analysis

Phylogeny for the species under study was obtained from TimeTree database [14] with branch length. Then, phylogenetically independent contrasts [15] analysis was performed with APE package [16] by executing pic function in R software (R version 3.2.3). After obtaining phylogenetically independent contrasts data of genomic variables and *Neu*, the matrix was tabulated and then subjected to the same multivariate statistical analysis as described in the above section.

### Results

**Fig. 1:**
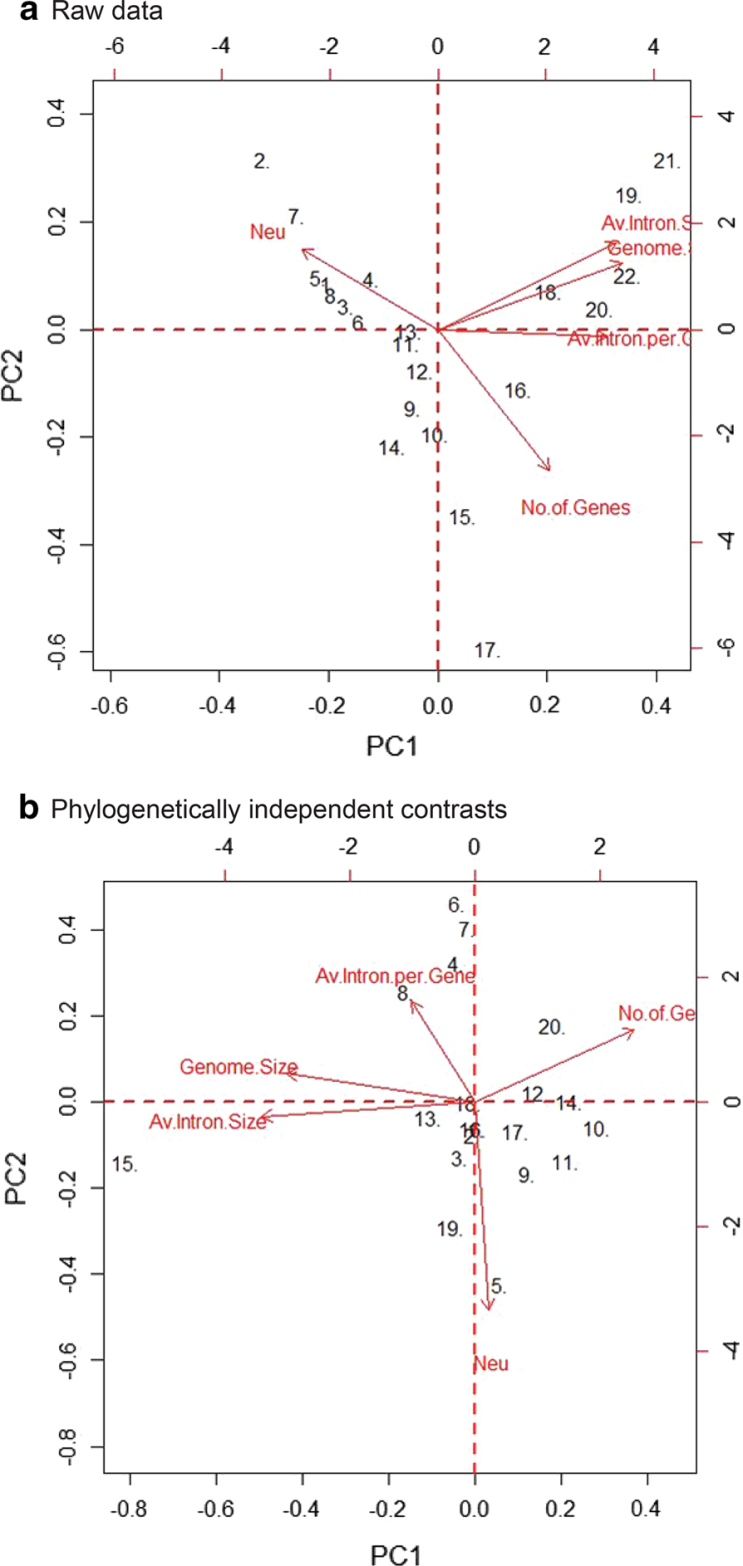
Principal component analysis of genomic variables and *Neu*. **a** PCA biplot from raw data. **b** PCA biplot from phylogenetically independent contrasts. The horizontal scale denoted principal component 1 (PC1) and the vertical scale denoted principal component 2 (PC2) in the biplot. The genomic variables are genome size in Mb (Genome.size), average intron size in bp (Av.intron.size), number of genes (No.of.gene), average introns per gene (Av.intron.per.gene), all marked in red. The effective population size and mutation rate (*Neu*) also marked in red. The red arrows showed the magnitude and direction of the genomics variables and *Neu* vectors

**Fig. 2:**
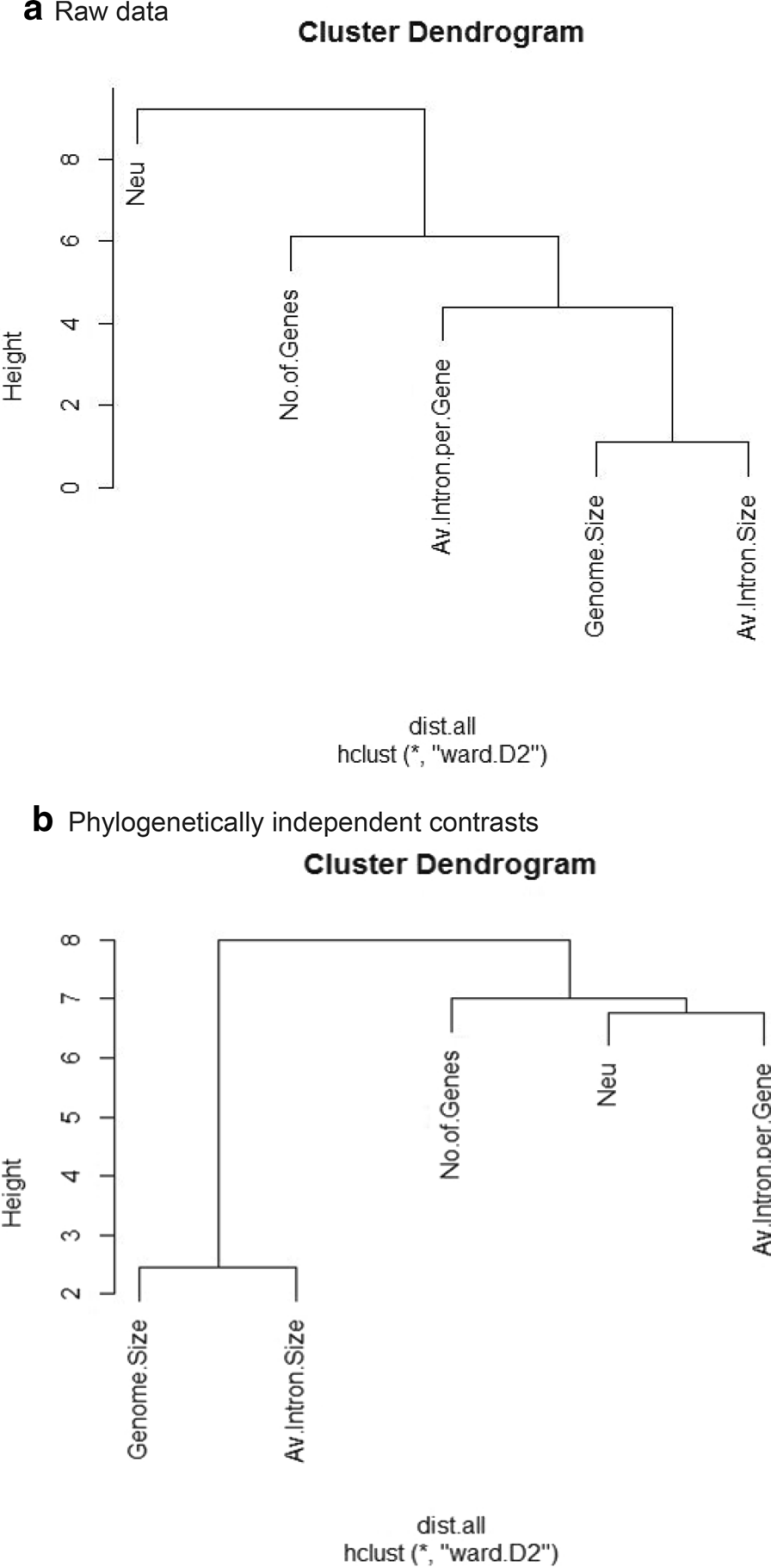
Cluster analysis of genomic variables and *Neu*. **a** A dendrogram obtained from hierarchical cluster analysis by using euclidean distance and ward.D2 methods on raw data. Two distinct clusters are formed around the height of eight. The *Neu* formed one cluster and genomics variables formed another cluster. **b** A dendrogram acquired by hierarchical cluster analysis after applying euclidean distance and ward. D2 methods on phylogenetically independent contrasts. Three distinct clusters are formed around the height of seven. The observations Genome size and Average intron size are quite similar as they formed cluster at the bottom of the dendrogram, while *Neu* formed cluster with Average introns per gene only

**Fig. 3:**
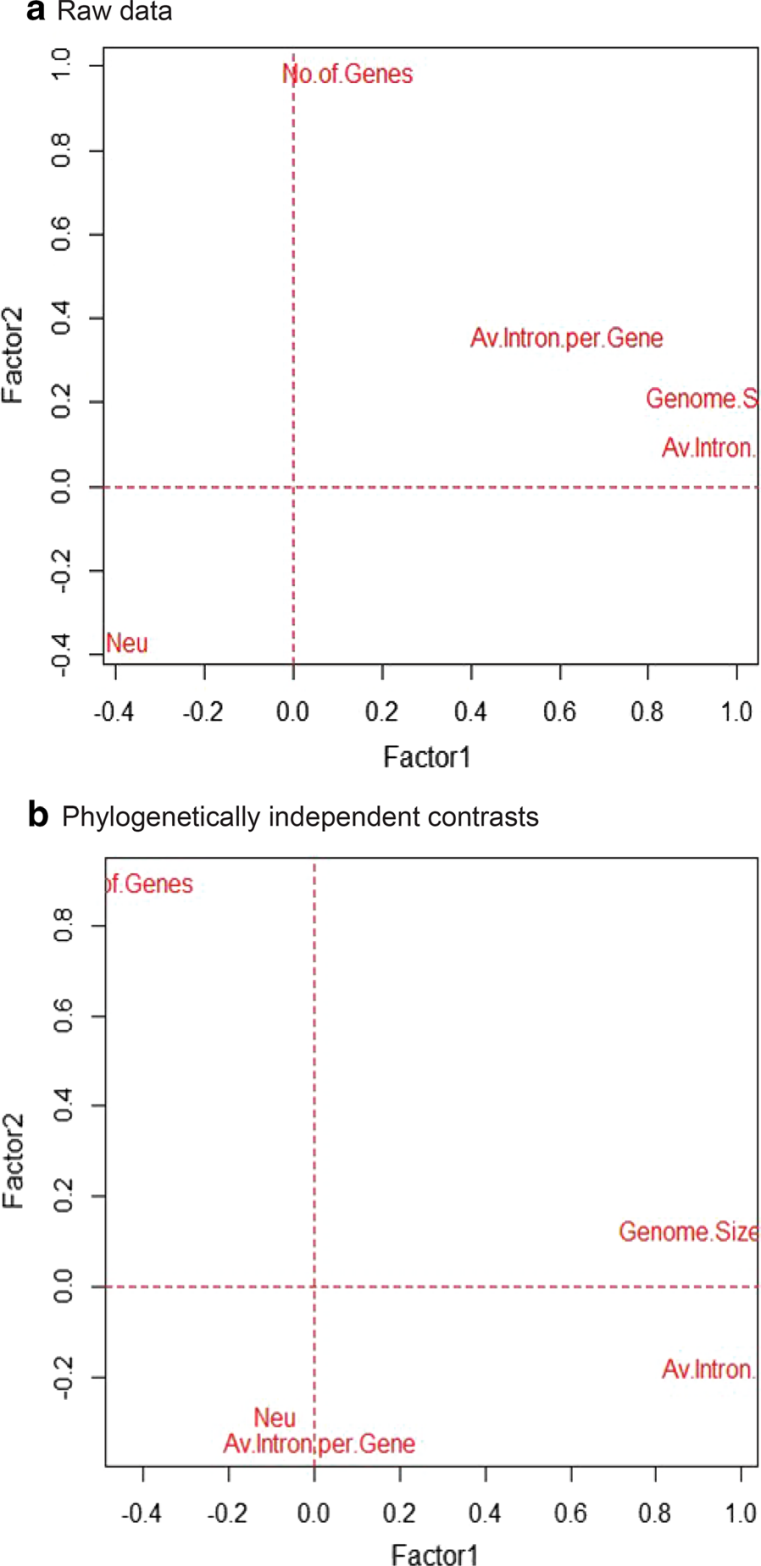
Exploratory factor analysis of genomic variables and *Neu*. **a** An EFA plot is generated from the standardized dataset by using factanal function and varimax rotation in R software on raw data. **b** An EFA plot is generated from phylogenetically independent contrasts by applying factanal function and varimax rotation. Two factors were considered as latent variables. The genomic variables and *Neu* are regarded as manifest variables. The genomic variables showed no relationships with *Neu* in raw data but in phylogenetically independent contrasts, only association between *Neu* and Average introns per gene is observed

We used raw data to compare with Lynch and Conery [1] analyses, while phylogenetically independent contrasts method was used to provide nonindependence of species in this study. We could not find relationship between genomic variables and *Neu* in PCA (Fig. 1a, b), CA (Fig. 2a, b), and EFA (Fig. 3a, b) by applying raw data but in phylogenetically independent contrasts, we found only association between intron number and *Neu*. Additionally, we did find relationship among genomic variables only.

The PCA analysis reduces the dimensionality of multivariate dataset and explains the variability pattern among multiple variables by showing most of the variation in all original variables as principal components (PC) [17]; therefore, we chose this method to summarise genomic variables and *Neu* to uncover any evolutionary patterns. The PC1 explained 61.24% and 44.06% of the variance, while PC2 explained an additional 19.21% and 23.91% of the variance in biplot for raw data and phylogenetically independent contrasts, respectively. Surprisingly, the angles between vector of genomic variables viz. genome size, average intron no per gene, average intron size, gene number, and *Neu* were wide and in different directions, suggesting there were no correlations in raw data (Fig. 1a). But in phylogenetically independent contrasts (Fig. 1b), we found angle between intron number and *Neu* to be narrow although they were in opposite directions, suggesting moderate inverse correlation. All vector lengths showed similar magnitude, suggesting almost equal contribution to the overall variance in the analysis. Similarly, we found correlation between genome size and intron size as indicated by small angle between these variable’s vectors in phylogenetically independent contrasts (Fig. 1b).

The cluster analysis is the most parsimonious way to cluster variables in term of correlation or distance in the multivariate dataset [17], therefore; we used this method to find if any relationship exists between genomic variables and *Neu*. However, in the cluster analysis, the genomic variables formed one cluster while *Neu* formed another cluster, indicating no similar variability pattern between them in raw data (Fig. 2a) but in phylogenetically independent contrasts, intron number and *Neu* clustered together (Fig. 2b). This clearly indicated that intron number tend to vary together with *Neu*. Similarly, genome size and intron size also formed cluster in phylogenetically independent contrasts (Fig. 2b).

Finally, we performed EFA analysis to disclose any hidden relationships among genomic variables and *Neu*. Perhaps *Neu* affects genome evolution by indirectly modulating genomic variables, and we may see some hidden relationships in the EFA analysis. By contrast, we clearly observed that genomic variables and *Neu* were not at the same location in the plot, revealing that there were no hidden relationships between them in raw data (Fig. 3a) but in phylogenetically independent contrasts (Fig. 3b), intron number and *Neu* were located at similar position in the plot, indicating association between them therefore we believe that intron evolution was modulated by *Neu*. In raw data, Factor 1 had the highest loading for average intron size than genome size, suggesting some correlation between these two variables, with 49% of total variance. In Factor 2, gene number had the highest loading compared to average intron no per gene, indicating relatively small correlation, but *Neu* had the lowest loading thus playing no part, with 26% of total variance. Similarly, in phylogenetically independent contrasts, Factor 1 had the highest loading for average intron size compared to genome size, suggesting some correlation, with 39% of total variance. In Factor 2, we found the highest loading for gene number but intron number and *Neu* had the lowest loading, implying some correlation, with 21% of total variance. In the current analysis, we deduced that *Neu* played almost no part in the evolution of genome size and complexity but may have influenced intron evolution.

### Discussion

One of the fundamental questions regarding genome size evolution is to examine if different genomic variables within genome vary together according to their correlations during evolution [18], and which evolutionary forces cause them to vary in such a correlative fashion is an unsolved mystery. Our multivariate analysis shows no relationship between genomic variables and *Neu* using only raw data but in phylogenetically independent contrasts, we observed some correlation between *Neu* and intron number. This observation is remarkably consistent with some results from the previous research [19]. But this observation is contrary to the previous conclusions which were based on the bivariate method only [1]. We found relationship between *Neu* and intron number by using phylogenetically independent contrasts method only. This shows that intron evolution may be influenced by *Neu*. Moreover, *Ne* in higher organisms is always smaller than that in prokaryotes; therefore, it is believed that introns never colonised the prokaryotic genome. Eukaryotes, however, fixed the introns in their genome and the average number of introns per gene increased with an increase in the complexity of organisms [20]. Interestingly, in agreement with the notion that introns were only fixed in eukaryotes, analysis of introns in cellulose synthase gene suggested that introns are eukaryotic invention [21]. A high *u* in *Arabidopsis* caused loss of introns, exemplifying the importance of *u* in intron and genome size evolution, as predicted by the mutational hazard hypothesis [22]. While analysis of non-recombining region of the genome as a site of inefficient selection showed no signs of introns gain. This contradicted the notion that a genetic drift alone was responsible for gain of introns in the multicellular organisms [23].

Other genomic variables such as genome size and intron size showed similar variability patterns with each other but not with *Neu*. In contrast, the analysis of a phylogenetically diverse group of species, genome size, average intron length, and *Neu* showed strong negative correlations, suggesting that random genetic drift plays a significant role in genome size evolution [1]. Equally our multivariate analysis contradicted this theory, as we can see *Neu* does not have relationship with either genome size or average intron size. Probably, a drift alone may not be responsible for the evolution of genome size. For instance, salamanders have large genome, and they exhibit a persistent long-term reduction in the population size. But there is no evidence of drift in their long evolutionary history. In this regard, the lower mutational hazard may have contributed to large genome size in these tetrapods as opposed to the mutational hazard hypothesis of genome size evolution [24]. In case of seed beetles, the reproductive fitness as a measure of selection was highly correlated with genome size which implies that natural selection has contributed to their genome size [25].

The variability pattern of gene number of an organism was not similar to *Neu*. However, a sufficient correlation between average gene number and *Neu* has already been established [1]. In many other eukaryotes, genome size has been found to show a positive correlation with gene number [26]. To date, genome sequencing in various taxa has revealed that gene number does not correlate with complexity of the organism in case of eukaryotes. For instance, humans are the most complex in terms of development, but they do not possess large number of genes than *Caenorhabditis elegans* [27].

A correlation exists between few phenotypic traits such as cell size, metabolic rate, developmental rate, and genome size [6]. Lynch and Conery [1] attempted to explain genome complexity by considering key population genetic parameter *Neu*, but received criticism because they could not consider phylogenetic relationships and their association with genomic variables in case of large phylogenies, and all genomic variables did not show a correlation with *Neu* [19]. The lack of association between genomic variables and *Neu* may be due to inaccuracy in time estimates of species divergence [28]. Although time estimates are controversial [29], but we think that phylogenetically independent contrasts data is more reliable than raw data since phylogenetic information is important to obtain meaningful conclusions. Here, we revisited Lynch and Conery dataset with a more holistic approach by multivariate analysis in order to determine any undisclosed patterns among genomic variables and *Neu*. Our results were not the same as the previous results obtained using the bivariate method only [1]. We confirmed that *Neu* may influence intron evolution in a correlative fashion but with no other genomic variables, implying that the enigma of genome size variation and organismal complexity needs further investigation.

## Limitations

1. We acknowledge that our study is based on analysis of previous data using multivariate methods to gain new insights into genome evolution. Here, we could only analyse eukaryotic genome because there are no introns present in the prokaryotic genome. This exclusion of prokaryotic data in this study provides only views regarding eukaryotic genome evolution.
2. The multivariate statistical analysis methods are exploratory methods, which analyse several variables together for the interpretation of the datasets. Thus, this method lacks quantitative measurements.

## Additional file

**Additional file 1.** A multivariate dataset of genomic variables and *Neu.* It is a table with species names and numerical values of genomic variables and *Neu.*

## Abbreviations

PCA: principal component analysis
CA: cluster analysis
EFA: exploratory factor analysis
*Ne*: effective population size
*u*: mutation rate
*Neu*: effective population size and mutation rate
