# Supplementary material for: Multivariate analysis of genomic variables, effective population size, and mutation rate
Source: BMC Res Notes. 2019 Jan 25;12:60. doi: 10.1186/s13104-019-4097-3 (PMC6347809; doi:10.1186/s13104-019-4097-3)
Supplement: Supplementary file 1 — Additional file 1. A multivariate dataset of genomic variables and Neu. It is a table with species names and numerical values of genomic variables and Neu. [file 13104_2019_4097_MOESM1_ESM.doc]

**Table S1.** A multivariate dataset of genomic variables and *Neu* [1, 8-13, NCBI annotation release101].

|  | Species | Genome Size (Mb) | Neu | No. of Genes | Av. Intron Size (bp) | Av. Intron per gene |
| --- | --- | --- | --- | --- | --- | --- |
| 1 | *Encephalitozoon cuniculi* | 2.9 | 0.00351 | 1997 | 38 | 0.0065 |
| 2 | *Saccharomyces cerevisiae* | 12.05 | 0.02294 | 6213 | 288 | 0.041 |
| 3 | *Schizosaccharomyces pombe* | 12.6 | 0.00294 | 4824 | 81 | 0.96 |
| 4 | *Chlamydomonas reinhardtii* | 120 | 0.01638 | 16790 | 336 | 6.30 |
| 5 | *Giardia lamblia* | 12.3 | 0.00759 | 5800 | 35 | 0.0002 |
| 6 | *Plasmodium falciparum* | 22.85 | 0.00082 | 5268 | 179 | 1.39 |
| 7 | *Dictyostelium discoideum* | 34 | 0.01825 | 9000 | 177 | 1.29 |
| 8 | *Neurospora crassa* | 42.9 | 0.0113 | 13000 | 101 | 1.6 |
| 9 | *Caenorhabditis elegans* | 100.26 | 0.00328 | 21200 | 124 | 4.94 |
| 10 | *Arabidopsis thaliana* | 125 | 0.00323 | 25498 | 170 | 6.18 |
| 11 | *Drosophila melanogaster* | 137 | 0.00374 | 13676 | 628 | 4.67 |
| 12 | *Ciona intestinalis* | 156 | 0.00305 | 16000 | 300 | 5.8 |
| 13 | *Anopheles gambiae* | 278 | 0.00298 | 13683 | 1061 | 3.47 |
| 14 | *Daphnia pulex* | 250 | 0.004674 | 31000 | 170 | 1.24 |
| 15 | *Fugu rubripes* | 365 | 0.00101 | 38000 | 298 | 5.2009 |
| 16 | *Clupea harengus* | 850 | 0.0008 | 24565 | 1617 | 7.89 |
| 17 | *Oryza sativa* | 466 | 0.00077 | 60256 | 446 | 3.6 |
| 18 | *Ficedula albicollis* | 1118 | 0.00092 | 16763 | 3242 | 6.34 |
| 19 | *Bos taurus* | 2725 | 0.0003589 | 21295 | 6002 | 4.20 |
| 20 | *Mus musculus* | 2500 | 0.00027 | 30000 | 3888 | 7.4 |
| 21 | *Pan troglodytes* | 3231 | 0.000288 | 23,534 | 7302 | 3.00 |
| 22 | *Homo sapiens* | 2900 | 0.00031 | 30000 | 4661 | 7.7 |
